# Age-Specific Pertussis Transmission Dynamics and Impact of Immunization Schedule Revision in Hunan, China: Age-Structured Mathematical Modeling Study

**DOI:** 10.2196/104376

**Published:** 2026-10-27

**Authors:** Yue He, Ziyan Liu, Yanhua Yang, Kangguo Li, Yao Wang, Yuqing Wu, Zheni Bu, Yuyang Liu, Yanhua Su, Qiuping Chen, Zeyu Zhao, Jia Rui, Kaiwei Luo, Tianmu Chen

**Affiliations:** 1 State Key Laboratory of Vaccines for Infectious Disease, Xiang An Biomedicine Laboratory, State Key Laboratory of Molecular Vaccinology and Molecular Diagnostics, School of Public Health, Xiamen University Xiamen, Fujian China; 2 Center for Disease Control and Prevention of Hunan Province (Hunan Academy of Preventive Medicine) Changsha, Hunan China; 3 School of Public Health, Chengdu Medical College Chengdu, Sichuan China; 4 Department of Traditional Chinese Medicine, Jinshan Sub District Community Health Service Center Xiamen, Fujian China; 5 Department of Epidemiology and Health Statistics, Xiangya School of Public Health, Central South University Changsha, Hunan China

**Keywords:** pertussis, whooping cough, vaccination, disease transmission, infectious, immunization programs, mathematical modeling, epidemiology, China

## Abstract

**Background:**

Despite extensive vaccination efforts, pertussis has experienced a global resurgence, with China alone reporting over 470,000 cases in 2024. The age distribution of cases has shifted markedly from infants to school-age children and adults, likely driven by waning acellular pertussis vaccine (DTaP [Diphtheria, Tetanus Toxoids, and Acellular Pertussis Vaccine])–induced immunity. In response, China revised its immunization schedule in 2025 to include a booster dose at the age of 6 years; however, the population-level impact of this strategy on age-specific transmission dynamics remains unclear.

**Objective:**

This study aimed to characterize the shifting age-specific transmission dynamics of *Bordetella pertussis* under changing immunization landscapes and evaluate the population-level impact of the 2025 Chinese immunization schedule.

**Methods:**

An age-structured Vaccinated-Susceptible-Exposed-Infectious-Recovered model incorporating vaccination, waning immunity, and demographic structures was calibrated using surveillance data from Hunan Province (2010-2025). The next-generation matrix approach estimated the effective reproduction numbers across 5 epidemic phases. Multiscenario simulations were used to assess the impact of a 6-year booster dose at varying coverage levels.

**Results:**

Among 47,300 reported cases (2010-2025), a marked resurgence peaked at 6743 cases in April 2024, with consistent spring seasonality. The case distribution shifted from infant-dominated (0-6 months: 293/552, 53.07%, 2010-2017) to school-age-dominated (6-10 years: 9814/30,589, 32.08%, 2024-2025). This study period was stratified into 5 temporal phases based on the epidemic dynamics: phase 1 (2010-2017), phase 2 (2018-2019), phase 3 (2020-2023), phase 4 (2024), and phase 5 (2025). Segmented fitting analyses were conducted for monthly pertussis cases across age groups within each phase of this study. The model demonstrated high fitting consistency across all age groups, with an overall *R*^2^ of 0.96. Adults emerged as the dominant transmission source in 2023-2024 (*R_eff_* to infants: 2.717). A booster administered at the age of 6 years with full coverage is expected to decrease the 10-year cumulative incidence by 24.15% (24,972.58/103,408.95). Children between 6 and 10 years of age are expected to experience the most significant monthly decrease, at 45.06% (335.49/744.51), whereas infants are predicted to gain 28.91% (170.92/591.19) indirect protection.

**Conclusions:**

Pertussis resurgence is driven by age-structured heterogeneity and waning immunity. Administering a booster shot to children at the age of 6 years can reduce the spread of infections across different age groups and offer indirect protection to infants who are at high risk. However, for long-term control, it may be necessary to implement broader strategies that involve vaccinating adolescents, adults, and pregnant women.

## Introduction

Pertussis, caused by *Bordetella pertussis*, is a highly contagious acute respiratory disease with a basic reproduction number (*R*_0_) estimated at 5.5-17 [1,2]. However, as vaccination coverage expands, the effective reproduction number (*R_eff_*)—which accounts for population immunity—becomes a more informative metric for tracking transmission dynamics under real-world immunization conditions. In China, pertussis is classified as a class B notifiable infectious disease, with children under 5 years of age constituting the primary susceptible population [3]. Among them, infants younger than 1 year are disproportionately affected, accounting for the highest incidence and the majority of severe cases [4]. Clinically, the disease is characterized by paroxysmal spasmodic cough with a distinctive inspiratory whoop, and the course of illness may last for 2-3 months [5,6]. Although the case fatality rate is low (0.01% in China during 2018-2021) [7], the disease burden remains considerable due to its high transmissibility and concentration in vulnerable populations, particularly in incompletely vaccinated infants [4].

From an epidemiological perspective, before the widespread use of vaccines, pertussis exhibited cyclical outbreaks occurring every 3-5 years [8]. This periodicity was substantially attenuated following the incorporation of pertussis vaccines into national immunization programs. However, since the early 21st century, a resurgence of pertussis has been reported in multiple regions worldwide, including countries with high vaccination coverage [9]. Similar trends have been observed in China, where the reported incidence has increased steadily since 2014, except for a temporary decline during the COVID-19 pandemic owing to nonpharmaceutical interventions (NPIs). Notably, reported cases rose sharply during the large 2024 outbreak, with more than 470,000 cases reported in China [8].

Concomitant with this resurgence, the age distribution of pertussis cases in China has substantially shifted [10]. Since 2017, the proportion of cases among adolescents and adults has increased markedly, resulting in a bimodal age distribution characterized by peaks in both infants and older age groups. This pattern suggests that, in addition to infants, older individuals may play an increasingly important role in sustaining transmission. Notably, this epidemiological trend intensified in 2024, when both China and multiple regions worldwide experienced large-scale pertussis outbreaks. Despite consistently high vaccination coverage exceeding 99% [3], severe disease remains concentrated among young infants who have not completed the vaccination schedule [6], while most cases in other age groups are mild and recover with appropriate treatment.

The observed resurgence and age shift of pertussis have been widely attributed to waning vaccine-induced immunity, pathogen adaptation, and improved surveillance and diagnostic capacities. Among these factors, the durability of vaccine-induced immunity is considered a key driver of changes in transmission dynamics [7,11,12]. In China, the pertussis vaccination strategy has evolved from DTwP (Diphtheria, Tetanus, and Whole-Cell Pertussis Vaccine) to DTaP (Diphtheria, Tetanus Toxoids, and Acellular Pertussis Vaccine), with progressive optimization of the immunization schedule. The incorporation of DTwP into the national immunization program in 1978, with a 4-dose schedule, led to a substantial decline in the disease incidence. The subsequent replacement with DTaP between 2007 and 2013 improved vaccine safety and acceptability but has been associated with more rapid waning of immunity [12,13], potentially contributing to increased susceptibility in older age groups [14].

To address these challenges, China further optimized its pertussis immunization schedule in 2025 by advancing the first DTaP dose to 2 months of age and introducing an additional booster dose at 6 years, resulting in a 5-dose regimen (2, 4, and 6 months; 18 months; and 6 years) [15]. This adjustment is intended to enhance early life protection and mitigate the role of school-age children in transmission. However, the population-level impact of this updated strategy, particularly its effects on age-specific transmission dynamics, remains unclear. Age-structured mathematical models, particularly the Vaccinated-Susceptible-Exposed-Infectious-Recovered (VSEIR) framework, have been widely applied to characterize pertussis transmission dynamics and evaluate vaccination strategies, with consistent evidence that waning immunity in older age groups sustains transmission toward vulnerable infants [16-20].

Building on this evidence, the present study pursued 3 specific objectives. First, we aimed to characterize the phase-dependent evolution of age-specific pertussis transmission dynamics in Hunan Province over the period 2010-2025 using a 5-phase segmented VSEIR modeling framework with next-generation matrix (NGM) analysis. Second, we sought to quantify the shifting contributions of different age groups, particularly older children, adolescents, and adults, to sustained pertussis transmission under progressively waning vaccine-induced immunity. Third, we evaluated the potential population-level impact of China’s 2025 immunization schedule revision, with a focus on the newly introduced booster dose at the age of 6 years, across a range of coverage scenarios and time horizons. Together, these analyses provide a quantitative basis for optimizing age-targeted pertussis vaccination strategies and informing future immunization policy in China.

## Methods

### Data Sources

This study used publicly available surveillance data from the Chinese Disease Surveillance System. No individual-level personal data were collected or used, and no human subjects were directly involved in this research. Therefore, ethical approval and informed consent were not required. All analyses were conducted in accordance with the Declaration of Helsinki.

Hunan Province, located in central China, was selected as the study area owing to its large demographically representative population, well-established infectious disease surveillance system, and high compliance with national immunization policies. Individual-level pertussis case records reported between January 2010 and December 2025 were obtained from the Hunan Provincial Center for Disease Control and Prevention. Each record included the date of illness onset and the patient’s age at diagnosis.

Age was recorded in months for children under 1 year of age and in years for individuals aged 1 year and older. Cases were aggregated into 6 age groups based on immunization schedule relevance and epidemiological characteristics: 0-6 months, 6 months-2 years, 2-6 years, 6-10 years, 10-20 years, and ≥20 years. These age boundaries were chosen to align with the Chinese national immunization schedule: 0-6 months corresponds to the window before completion of the primary DTaP series (doses at 2, 4, and 6 months); 6 months-2 years spans the period from completion of the primary series through the 18-month booster dose; 2-6 years represents the preschool period; 6-10 years isolates the cohort eligible for the booster dose introduced at the age of 6 years in 2025; 10-20 years covers adolescence; and ≥20 years represents adults. This definition ensures that each age group corresponds to a distinct vaccination status, enabling the model to directly capture the epidemiological impact of changes in the immunization schedule.

The 2025 immunization schedule revision encompassed 2 modifications: advancing the primary series from 3, 4, and 5 months to 2, 4, and 6 months of age, and introducing an additional booster dose at 6 years of age. This study focused exclusively on evaluating the epidemiological impact of the booster dose at 6 years of age, which targets an age group of increasing importance in sustaining pertussis transmission.

### Descriptive Analysis

Temporal trends in monthly reported cases and age-specific incidence rates were examined across the study period. Age-specific incidence rates were calculated using population structures derived from China’s national population censuses conducted at 5-year intervals. For each census year, age-specific population counts reported at 5-year age bands were used to construct a reference population structure, with uniform distribution assumed within each 5-year interval. Annual provincial population totals were obtained from the Hunan Provincial Statistical Yearbook, and age-specific population denominators were estimated by applying the census-derived proportional age structure to the corresponding annual total population figures. Intercensal years were handled by applying the population structure from the most recent preceding census.

To characterize the seasonal distribution of pertussis incidence, calendar months were classified into 4 meteorological seasons: spring (March-May), summer (June-August), autumn (September-November), and winter (December-February). For each season, cases were aggregated across all observation years, and the annual mean monthly incidence was estimated by dividing the cumulative case count by the corresponding number of observation years. Age-specific seasonal patterns were subsequently examined by computing the annual mean case count stratified by age group, season, and epidemic phase, with phase-specific denominators applied to account for the varying duration of each phase.

The study period (January 2010-December 2025) was divided into 5 phases based on key epidemiological transitions and national policy events. Phase 1 (2010-2017) corresponds to a prolonged period of relatively low pertussis incidence following the introduction of DTaP vaccination. Phase 2 (2018-2019) marks the onset of a marked resurgence, coinciding with the increased application of polymerase chain reaction (PCR) and serological PT-IgG antibody testing for laboratory confirmation [21]. Phase 3 (2020-2023) covers the COVID-19 pandemic period, during which NPIs substantially suppressed pertussis transmission [22]. Phase 4 (2024) captures the post-NPI relaxation period characterized by a historic outbreak peak, and phase 5 (2025) reflects the implementation of the revised national immunization schedule that introduced a 6-year booster dose.

Throughout the study period, pertussis was reported through the standardized National Notifiable Infectious Disease Reporting System. The diagnostic criteria followed the national standard WS 274, with the 2019 revision (WS 274-2019) adding PCR-based confirmation alongside clinical and serological criteria. These criteria were applied uniformly across all age groups and throughout the study period in Hunan Province, with no province-specific protocol changes that would introduce discrete discontinuities at the phase boundaries.

To objectively validate the phase definitions, we conducted change point detection analyses using 3 established algorithms, Pruned Exact Linear Time, cumulative sum, and Bayesian information criterion, applied independently to monthly case counts for each of the 6 age groups. Full results are presented in Figure S1A-E and Table S1 in Multimedia Appendix 1.

To assess whether sex-specific stratification would inform the transmission model, sex-stratified descriptive analyses of case counts, incidence rates, and age at onset, together with formal tests for sex differences, were performed as exploratory analyses (Figure S2A-D and Table S2 in Multimedia Appendix 1).

### Model Structure

An age-structured VSEIR compartmental model was developed to characterize pertussis transmission dynamics. The population was stratified into 6 age groups aligned with the national immunization schedule: 0-6 months (group 1), 6 months-2 years (group 2), 2-6 years (group 3), 6-10 years (group 4), 10-20 years (group 5), and ≥20 years (group 6). Within each age group, individuals were classified into 5 mutually exclusive epidemiological compartments: susceptible (S), vaccinated with waning immunity (V), exposed (E), infectious (I), and recovered (R).

The model incorporates age-specific demographic processes, including live births entering group 1, natural mortality, and aging transitions between adjacent groups at constant rates. Disease-induced mortality was not modeled given the negligible case fatality rate of pertussis. Population closure was assumed, with no migration (Figure 1).

**Figure 1:**
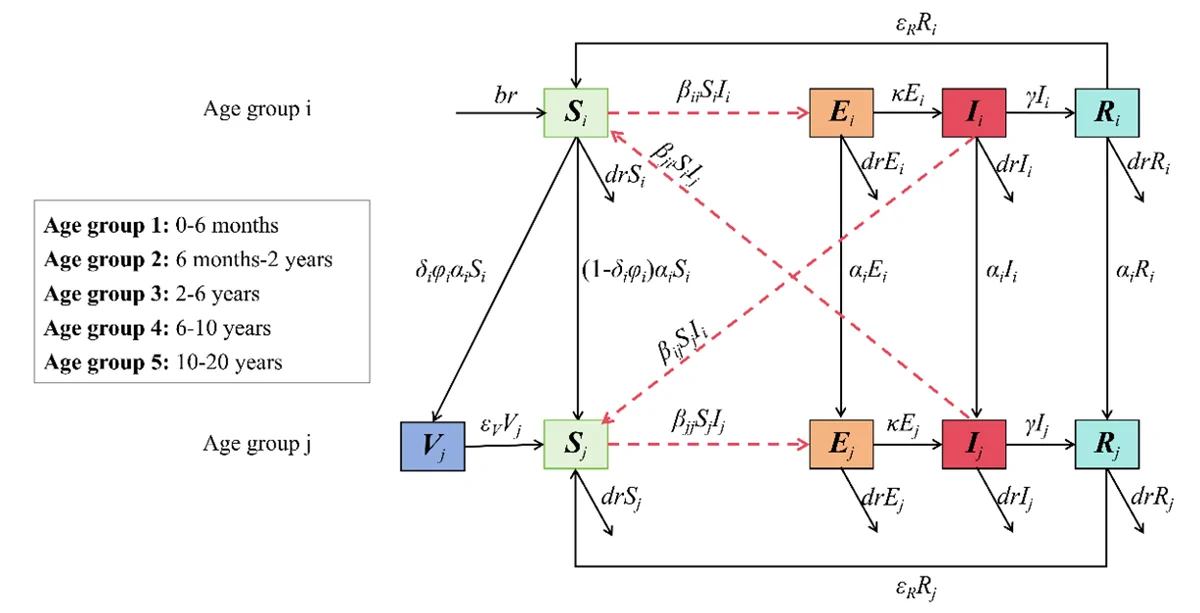
Structure of the age-structured VSEIR transmission model for pertussis. The population in each age group (i,j) is divided into 5 compartments: susceptible (S), vaccinated but not fully protected (V), exposed (E), infectious (I), and recovered (R). VSEIR: Vaccinated-Susceptible-Exposed-Infectious-Recovered.

1. Force of infection: Transmission follows a frequency-dependent mechanism. The force of infection on age group i is:

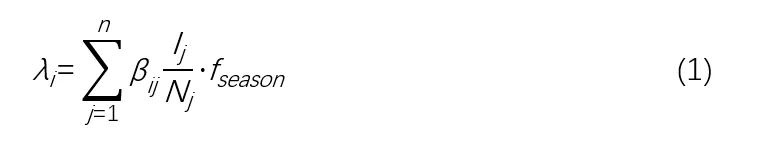



Where *β*_ij_ represents the transmission rate from age group *j* to age group *i*, I_j_(t) denotes the number of infectious individuals in group *j*, and N_j_(t) is the total population in group j. The term f_season_(t) captures seasonal variation in transmission.

2. Latent and infectious periods: Exposed individuals are noninfectious and transition to the infectious compartment at rate κ (mean latent period 1/κ). Infectious individuals recover at rate γ (mean infectious period 1/γ).

3. Waning immunity: Postinfection immunity wanes at rate *ε_R_*, vaccine-derived immunity wanes at rate *ε_V_*, returning individuals to the susceptible compartment. This reflects the incomplete and temporary nature of pertussis immunity.

4. Vaccination: Vaccination is modeled from group 2 onward. Age-specific vaccination coverage (φ_i_) and vaccine efficacy (δ_i_) jointly determine the proportion of individuals entering the vaccinated compartment upon aging into each group. Coverage for the 6-year booster, administered upon entry into group 4 (6-10 years), is the primary parameter modified in scenario analyses.

5. Reporting: An age-group-specific reporting rate ρ_i_ links model output to surveillance data. Reported incidence is defined as:



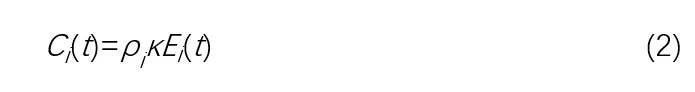



6. Phase-specific parameters: The study period is partitioned into 5 fitting phases, within which transmission parameters are assumed constant and estimated independently.

The complete differential equation system for each age group is presented below. For group 1 (0-6 months):



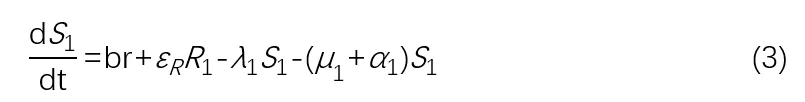





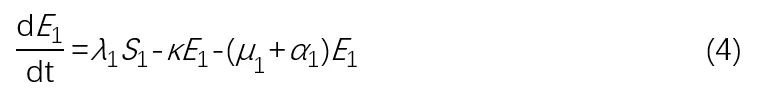





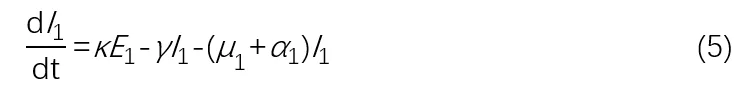





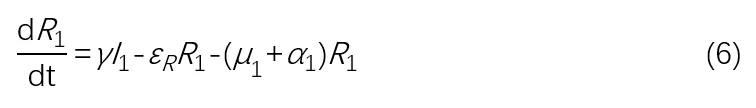



For group 2 (6 months-2 years):



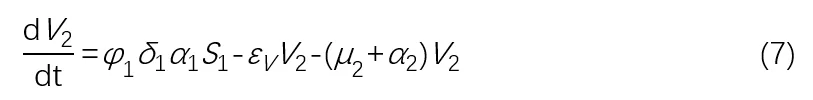





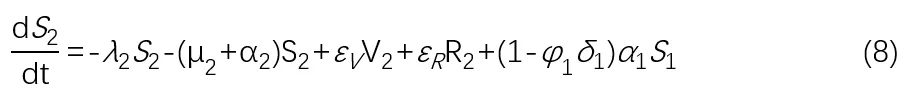





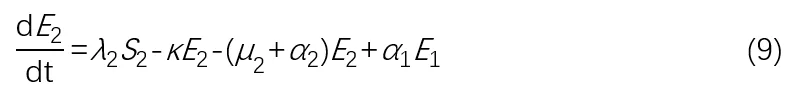





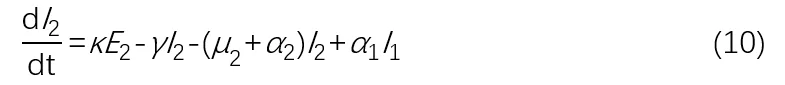





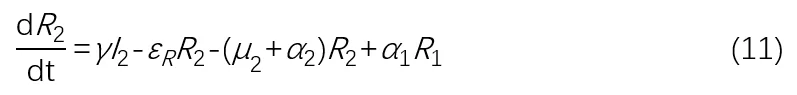



For groups 3-5 (*i*=3,4,5), the transition equations follow an analogous structure:



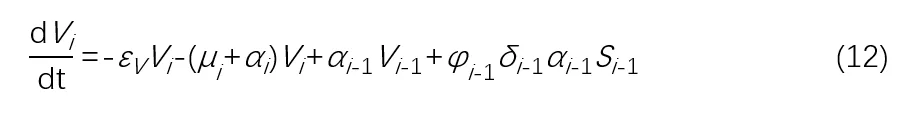





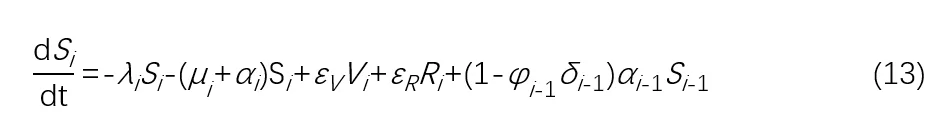





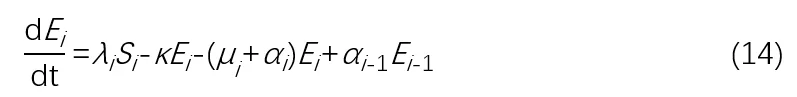





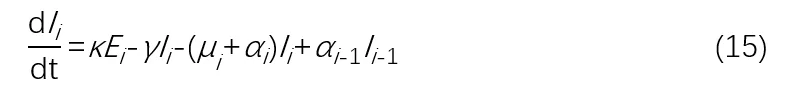





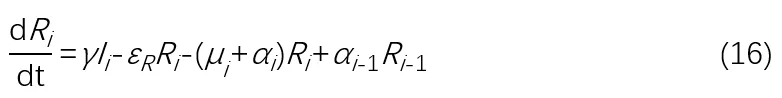



For group 6 (≥20 years), the aging-out term is omitted:



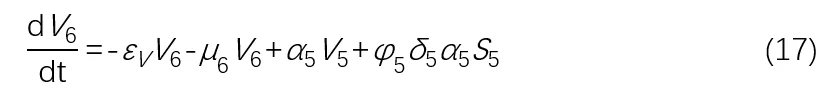





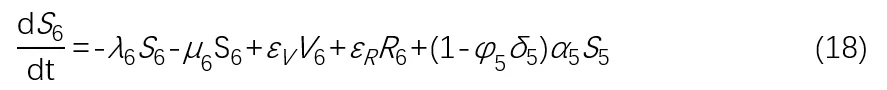





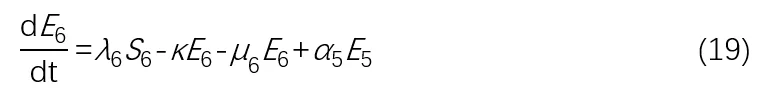





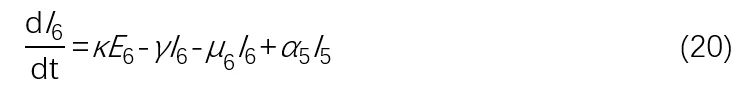





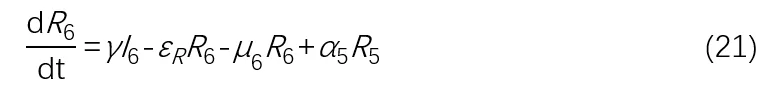



Natural history parameters (κ, γ, ε_R, _*ε_V_*) were informed by published literature, and demographic parameters including age-specific mortality and aging rates were derived from national census data. All fixed parameters and their sources are summarized in Table 1.

**Table 1:** VSEIR^a^ model parameters.

Parameter	Description	Unit	Value	Source
*β_ij_*	Transmission rate	person^-1^*month^-1^	—^b^	Fitting
*f_season_*	Seasonal modulation factor	1	—	Fitting
*N*	Population size (age group i)	1	Summed	Statistical Yearbook [23]
*br*	Number of newborns	month^-1^	Summed	Statistical Yearbook [23]
*μ_i_*	Natural mortality rate	month^-1^	Summed	Statistical Yearbook [23]
*ρ_i_*	Case reporting rate	1	—	Fitting
*α_i_*	Aging rate (transition between age groups)	month^-1^	1/6，1/18，1/48，1/60	Reported data
*κ*	Progression rate from exposed to infectious	month^-1^	3	US Centers for Disease Control and Prevention [24]
*γ*	Recovery rate (inverse of infectious period)	month^-1^	30/35	Finger and von Koenig [25]
*δ*	Projected intervention effectiveness	1	0.95, 0.90, 0.85	Decker and Edwards, Wilkinson et al, Zhu et al, Gambhir et al, Chit et al, Zhang et al [5,26-29]
*φ_i_*	Projected vaccination coverage rate	1	0.99, 0.73	Zhu et al, Zhang et al [27,30]
*ε_V_*	Vaccine-induced immunity waning rate	month^-1^	1/60	Chen and He [11]
*ε_R_*	Natural immunity waning rate	month^-1^	1/120	Wendelboe et al [31]

^a^VSEIR: Vaccinated-Susceptible-Exposed-Infectious-Recovered.

^b^Not available.

### Model Fitting

Model parameters were estimated independently within each of 5 consecutive fitting phases (January 2010-December 2025) using a bounded nonlinear least-squares algorithm. Within each phase, the estimated parameter vector comprised a 6 × 6 age-structured transmission rate matrix *B*= [*β_ij_*] and age-group-specific reporting rates *ρ*= (*ρ*_1_, *ρ*_2_, *ρ*_3_, *ρ*_4_, *ρ*_5_, *ρ*_6_). The objective function minimized weighted squared residuals between model-predicted and observed monthly incidence across all 6 age groups simultaneously, with observation weights defined as a monotone function of phase-mean incidence to down-weight groups with extreme case counts.

Reporting rates were regularized through 2 penalty components. A Beta-distribution prior was applied to constrain reporting rate estimates toward biologically plausible values. For phases 2-5, an additional smoothing penalty was imposed to prevent abrupt discontinuities between adjacent phases, while preserving flexibility to capture genuine shifts in surveillance sensitivity. The seasonal forcing amplitude was specified independently for each phase.

Model fitting was implemented sequentially across phases. For phase 1, initial compartment sizes were constructed heuristically from observed incidence at the start of the study period and age-specific vaccination coverage assumptions. For phases 2-5, the terminal compartment state of the preceding fitted phase was carried forward as the initial condition for the subsequent phase, ensuring demographic and immunological continuity across phase boundaries. The ordinary differential equation system was solved numerically using the RK45 algorithm with strict convergence tolerances, and state variables were constrained to nonnegative values throughout integration. Model fit was evaluated using the coefficient of determination (*R*^2^) computed separately for each age group. For projection analyses, transmission parameters, reporting rates, and the terminal compartment state estimated from phase 5 were carried forward to simulate pertussis dynamics from January 2026 onward.

### Estimation of the Effective Reproduction Number

The effective reproduction number Reff was estimated for each fitting phase using an NGM framework adapted to incorporate age-specific susceptibility [32]. For phase k, the age-structured NGM K^(k)^ was constructed as:



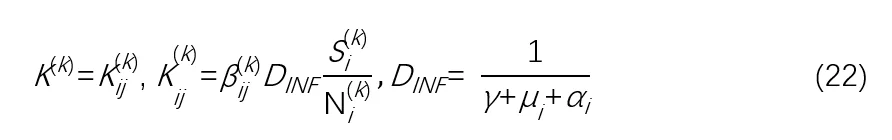



Where 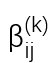 is the transmission rate from age group j to i in phase k, 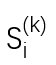 denote the susceptible population and total population of age group i, respectively. D_INF_ represents the mean infectious period. The effective reproduction number for phase k is calculated as the spectral radius of the matrix K^(k)^:



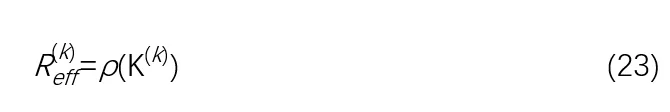



This formulation explicitly incorporates both age-specific transmission heterogeneity (through β_ij_), and time-varying susceptibility structure (through 
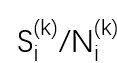
), ensuring mathematical consistency between matrix-level visualization and overall epidemic potential.

### Sensitivity Analysis

Sensitivity analyses were conducted to evaluate the robustness of transmission estimates and vaccine impact projections to uncertainty in case ascertainment and immunity-related parameters.

To assess parameter identifiability and the robustness of projections, we performed a parametric bootstrap for phases 4 and 5. Synthetic monthly case counts were generated from the fitted phase-specific incidence curves (negative binomial), and the full estimation procedure was repeated under the original constraints for 30 replicates. The replicate parameter sets were used to derive bootstrap 95% CIs for the effective reproduction number, aggregate transmission measures, and projected outcomes (Tables S2 and S3 in Multimedia Appendix 1).

To examine the influence of age-dependent underreporting on inferred transmission dynamics, the phase 4 NGM was recalculated under 4 alternative ascertainment scenarios. Because pertussis cases in older individuals are more likely to present with mild or atypical symptoms and therefore remain undetected, age-specific reporting rates were progressively reduced across older age groups relative to the fitted baseline values. The baseline scenario assumed no additional adjustment, whereas the remaining scenarios imposed increasingly stronger reporting gradients, with adult multipliers decreasing to 0.25, 0.12, and 0.08, respectively. For each scenario, the effective reproduction number and major age-specific transmission pathways were re-estimated and compared.

Sensitivity analyses for vaccine impact projections were performed using a full-factorial design across 3 key parameters, including vaccine-derived immunity duration of 3, 5, and 10 years, natural immunity duration of 5, 10, and 20 years, and 6-year booster vaccine efficacy of 71%, 85%, and 95%. All parameter combinations were evaluated under 6 booster coverage scenarios ranging from 0% to 100%, resulting in 162 independent simulation runs. Projected intervention effectiveness was defined as the proportional reduction in cumulative reported cases over a 10-year projection period relative to the 0-coverage baseline. Uncertainty bounds for each coverage scenario were derived from the full range of simulated outcomes across all parameter combinations.

### Vaccine Impact Projection

To evaluate the potential epidemiological impact of the 6-year booster dose introduced under the 2025 immunization schedule revision, projection analyses were conducted from January 2026 onward using parameters estimated from the final fitting phase. The baseline scenario assumed 0 booster coverage, representing the counterfactual in which the additional dose is not implemented. Five intervention scenarios were defined with booster coverage levels of 30%, 50%, 70%, 80%, 85%, 90%, 95%, 99%, and 100% applied to the 6-year-old cohort entering group 4, with all other model parameters held constant. The 100% coverage scenario represents a theoretical upper bound included to characterize the maximum achievable impact under ideal conditions.

For each scenario, projected intervention effectiveness was quantified as the proportional reduction in cumulative projected reported cases relative to the baseline, calculated both overall and separately for each age group. Short-term (3-year) and long-term (10-year) effectiveness estimates were derived to characterize the temporal dynamics of impact. Monthly peak case reduction was additionally calculated as the proportional difference in maximum monthly projected incidence between the 0% and 100% coverage scenarios within each age group.

### Statistical Analysis and Visualization

Percentages for projected scenarios represent relative reductions in the cumulative number of reported cases, computed for each parameter combination as (N0% – Nscenario)/N0%, where N0% is the projected cumulative case count under 0% booster coverage. Values reported for the sensitivity analysis are means across the specified parameter combinations, with the range across combinations given in parentheses. Descriptive analyses and data visualization were performed in R (version 4.5.1). Model simulations and projection analyses were implemented in Python (version 3.12.4).

### Ethical Considerations

The data were obtained from the Chinese Information System for Disease Control and Prevention, within the Hunan Provincial Center for Disease Control and Prevention’s (Hunan Provincial Academy of Preventive Medicine) surveillance project on key acute respiratory infectious diseases, which is one of the funding sources of this work. The source data in the national surveillance system contain personal identifiers; an exemption from ethics review was therefore not applicable under Chinese regulations, and formal ethics review was mandatory. Because personal identifiers were removed from the extracted records during the analysis phase and only de-identified records were analyzed, and because the study involved no direct patient involvement, medical intervention, or biological samples, the Ethics Review Committee of the Hunan Provincial Center for Disease Control and Prevention exempted the authors from obtaining informed consent. The study was reviewed retrospectively, after the data had already been collected, and was approved by the committee (approval 湘疾控IRD-2025-013-01; October 20, 2025). All results are reported as aggregated, population-level counts; no individual-level or identifiable data are presented in the manuscript or in any publicly available material.

## Results

### Overall Temporal Trend

Between 2010 and 2025, a total of 47,300 pertussis cases were reported in Hunan Province. Age-specific case distributions included: 0-6 months (n=8994), 6 months-2 years (n=7747), 2-6 years (n=13,576), 6-10 years (n=12,477), 10-20 years (n=2909), and ≥20 years (n=1597). Monthly reported cases and age-specific incidence rates (Figure 2A and B) revealed distinct temporal patterns. During 2010-2017, monthly reported cases were maintained between 0 and 30, with an age-specific incidence rate of 2.18 per 100,000 in 2017. A minor resurgence occurred in 2018-2019, with peak monthly cases reaching 271. During 2020-2022, monthly cases remained below 92. From 2023 onward, cases increased synchronously across all age groups, reaching the study-period peak in April 2024, with 6743 total monthly cases (age-group specific: 578, 647, 2197, 2472, 606, and 243, respectively). By late 2025, monthly reported cases had fallen below 56.

Age composition of cases demonstrated marked temporal variation (Figure 2C and D). During 2010-2017, cases predominantly concentrated in the 0-6 months age group (293/552, 53.07%), followed by 6 months-2 years (170/552, 30.79%) and 2-6 years (69/552, 12.50%), with other age groups each accounting for <2.00%. In 2018-2019, the proportion of 0-6-month cases decreased to 39.05% (2526/6469), while proportions of 6 months-2 years and 2-6 years groups increased to 38.21% (2472/6469) and 17.39% (1125/6469), respectively. During 2020-2023, the 0-6 months proportion further declined to 24.63% (2387/9690), whereas 2-6 years and 6-10 years groups increased to 28.73% (2784/9690) and 24.57% (2381/9690), respectively. In 2024-2025, younger age groups showed markedly reduced case proportions: 0-6 months (3788/30,589, 12.38%), 6 months-2 years (3604/30,589, 11.78%); while proportions in 2-6 years (9598/30,589, 31.38%), 6-10 years (9814/30,589, 32.08%), 10-20 years (2413/30,589, 7.89%), and ≥20 years (1372/30,589, 4.49%) increased substantially.

**Figure 2:**
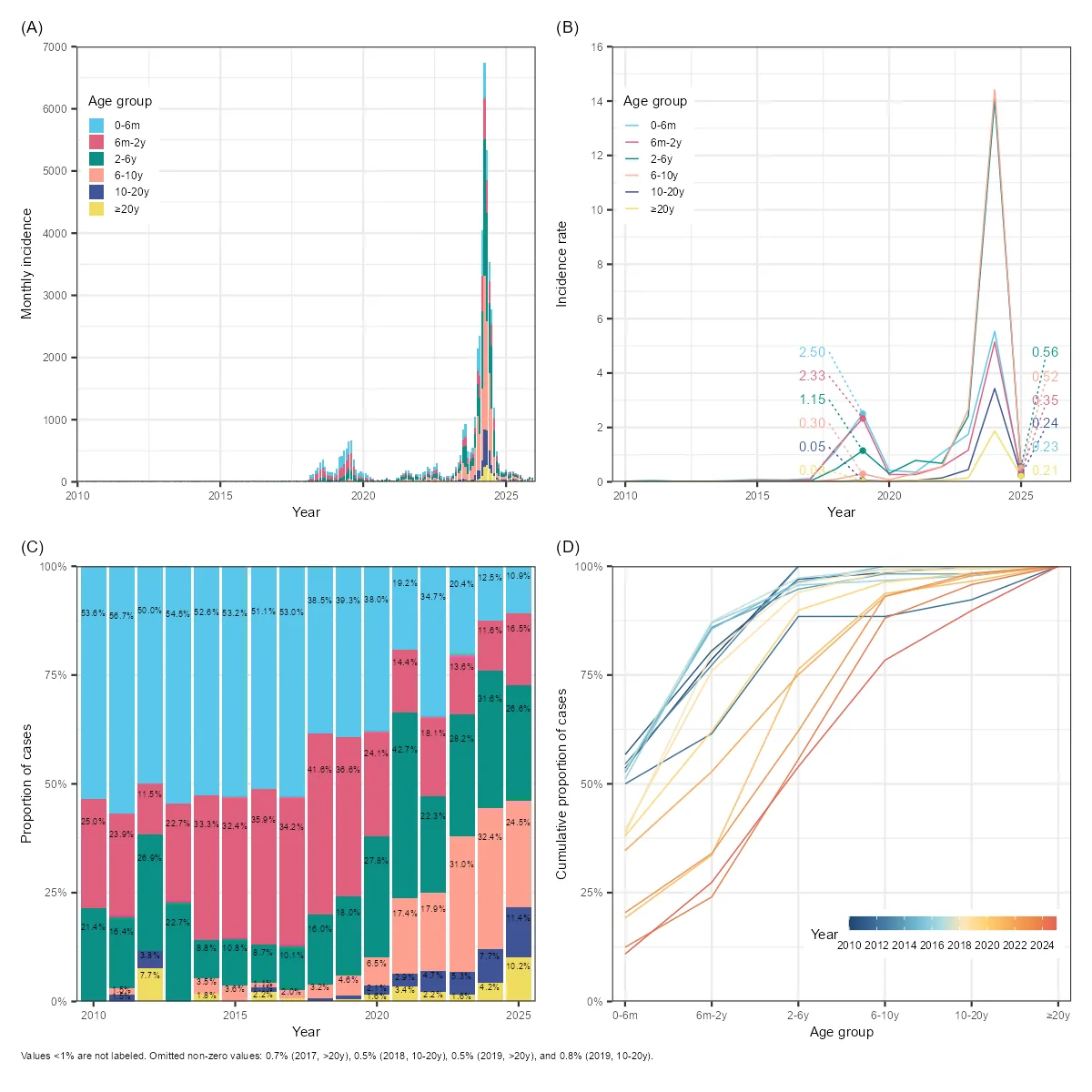
Pertussis incidence in Hunan Province, 2010-2025. (A) Cumulative reported cases by age group; (B) annual trend of age-specific incidence rates (annual cases per age group divided by total population in the corresponding year); (C) annual age composition of cases; and (D) cumulative age distribution of reported cases by year (2010-2024).

### Seasonal Distribution

Pertussis incidence in Hunan Province demonstrated a consistent seasonal pattern throughout the study period. The highest monthly case burden was recorded in April (8087 total cases across all years), with spring (March-May) representing the primary epidemic season, followed by a secondary peak in June-July. The lowest incidence occurred in autumn and winter, particularly in October and November (Figure 3A).

This spring-dominant pattern was maintained across all epidemic phases, though the age-group contributions varied markedly. During 2010-2017, the 0-6 months group contributed the highest spring burden (annual mean: 13.2 cases, SD 12.8 cases; Figure 3B). During 2018-2019, the 6 months-2 years group emerged as the dominant contributor in summer (annual mean: 574.5 cases, SD 219.9 cases; Figure 3C). During 2020-2023, summer became the peak season for most age groups, with the 2-6 years group showing the highest summer burden (annual mean: 275.0 cases, SD 287.9 cases; Figure 3D). During 2024, the 2-6 years group recorded the highest summer burden (2560 cases), while the 6-10 years group contributed substantially in spring (5383 cases; Figure 3E). During 2025, the 6-10 years group recorded the highest spring burden (126 cases), while the 2-6 years group contributed substantially in winter (120 cases; Figure 3F). Across all phases, autumn consistently represented the low-transmission season.

**Figure 3:**
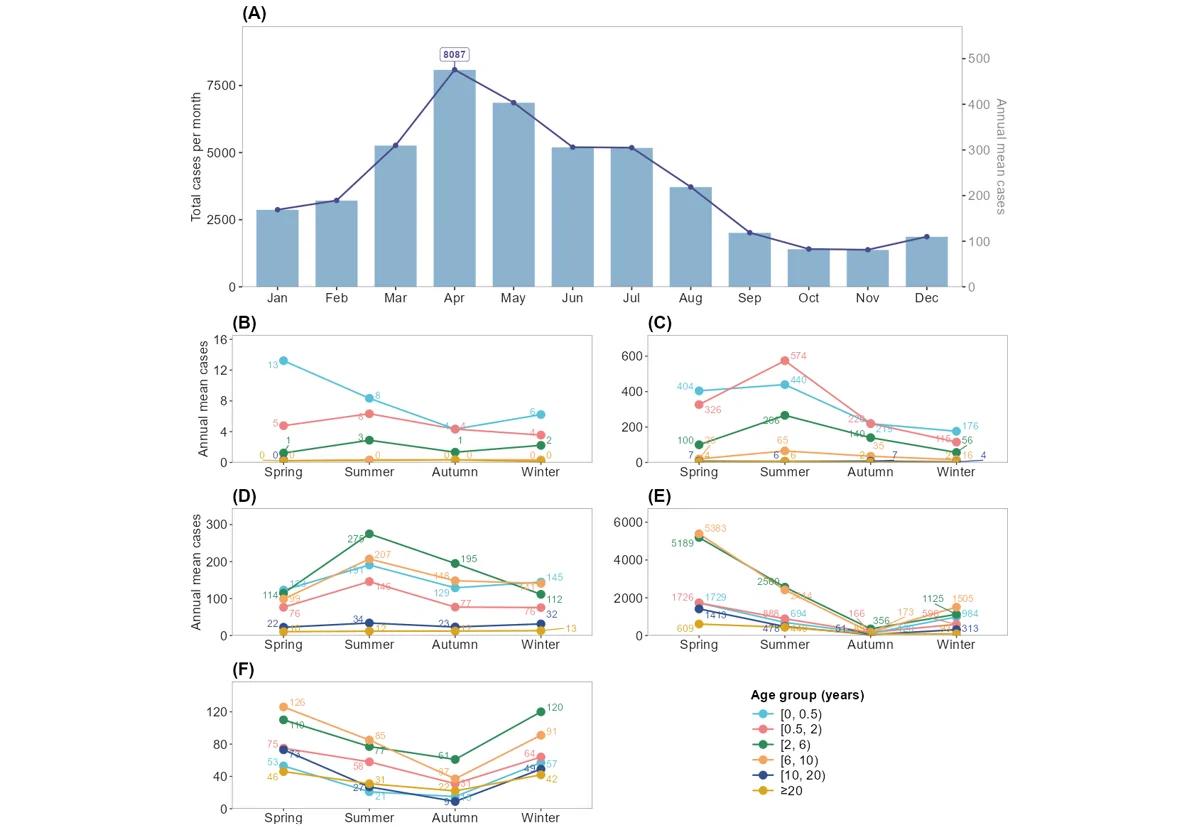
Seasonal distribution of pertussis cases across age groups in Hunan Province. (A) Total monthly cases and annual mean cases by calendar month. (B) Seasonal variation in annual mean cases by age group during phase 1 (2010-2017). (C) Seasonal variation in annual mean cases by age group during phase 2 (2018-2019). (D) Seasonal variation in annual mean cases by age group during phase 3 (2020-2023). (E) Seasonal variation in annual mean cases by age group during phase 4 (2024). (F) Seasonal variation in annual mean cases by age group during phase 5 (2025). In panels (B-F), seasons are defined as spring (March-May), summer (June-August), autumn (September-November), and winter (December-February).

### Model Performance

The study period was stratified into 5 temporal phases based on epidemic dynamics: phase 1 (2010-2017), phase 2 (2018-2019), phase 3 (2020-2023), phase 4 (2024), and phase 5 (2025). Segmented fitting analyses were conducted for monthly pertussis cases across age groups within each phase (Figure S3 in Multimedia Appendix 1). Groups 1-6 corresponded to ages 0-6 months, 6 months-2 years, 2-6 years, 6-10 years, 10-20 years, and ≥20 years, respectively. The model demonstrated high fitting consistency across all age groups, with coefficients of determination (*R*^2^) of 0.86 (group 1), 0.88 (group 2), 0.95 (group 3), 0.94 (group 4), 0.95 (group 5), and 0.82 (group 6). The overall *R*^2^ was 0.96.

Local identifiability diagnostics indicated that individual age-specific transmission coefficients were not fully identifiable in the short phases 4 and 5 (condition numbers of J’J on the order of 10^13^-10^14^ and near-0 eigenvalues for 29/42 and 19/42 parameters, respectively; Table S3 in Multimedia Appendix 1). In the parametric bootstrap, aggregate measures were more stable: the effective reproduction number was 1.24 (95% CI 0.92-1.86) in phase 4 and 1.15 (95% CI 0.73-1.40) in phase 5. Propagating the bootstrap parameter sets through the projection model yielded a 10-year reduction in reported cases of 20% (95% CI 11.3%-65.1%) at 70% coverage, with effectiveness increasing monotonically with coverage (Figure S4 and Table S4 in Multimedia Appendix 1).

### Transmissibility Dynamic Analysis

Cross-age-group *R_eff_* demonstrated phase-dependent shifts in pertussis transmission patterns. During phase 1 (2010-2017), transmission was concentrated among younger age groups, with the 6 months-2 years group recording the highest within-group *R_eff_* (1.039; Figure 4A). In phase 2 (2018-2019), within-group transmissibility in the 6 months-2 years group increased to 1.234, with elevated cross-age transmission from the 10-20 years to 6-10 years group (*R_eff_* = 0.510; Figure 4B).

During phase 3 (2020-2023), the highest cross-age *R_eff_* shifted to transmission from the 10-20 years to 6 months-2 years group (0.895), followed by 10-20 years to 6-10 years transmission (0.669; Figure 4C). In phase 4 (2024), transmission from the ≥20 years group became predominant, with *R_eff_* reaching 2.717 to the 6 months-2 years group and 2.050 to the 10-20 years group (Figure 4D). By phase 5 (2025), a bimodal transmission pattern emerged: the ≥20 years to 6-10 years pathway showed the highest *R_eff_* (1.603), while the 6 months-2 years to 6-10 years pathway also increased substantially (*R_eff_* = 1.411; Figure 4E). Concurrently, monthly reported cases declined to below 56 by late 2025.

The overall transmission matrix for the full study period (2010-2025) showed that transmission was primarily concentrated among school-age children and older age groups (Figure 4F). The strongest transmission pathways included within-group transmission in the 6 months-2 years group (*R_eff_* = 0.463), transmission from 10-20 years to 6-10 years (*R_eff_* = 0.593), and transmission from ≥20 years to 6-10 years (*R_eff_* =0.412). Cross-age transmission from older age groups to younger children remained consistently evident across the aggregated matrix.

Based on estimated transmission parameters, we projected pertussis incidence over the next decade (2026-2035) under 6 booster vaccination coverage scenarios at the age of 6 years: 0%, 30%, 50%, 70%, 90%, and 100%, assuming vaccine efficacy per dose remains at historical levels.

**Figure 4:**
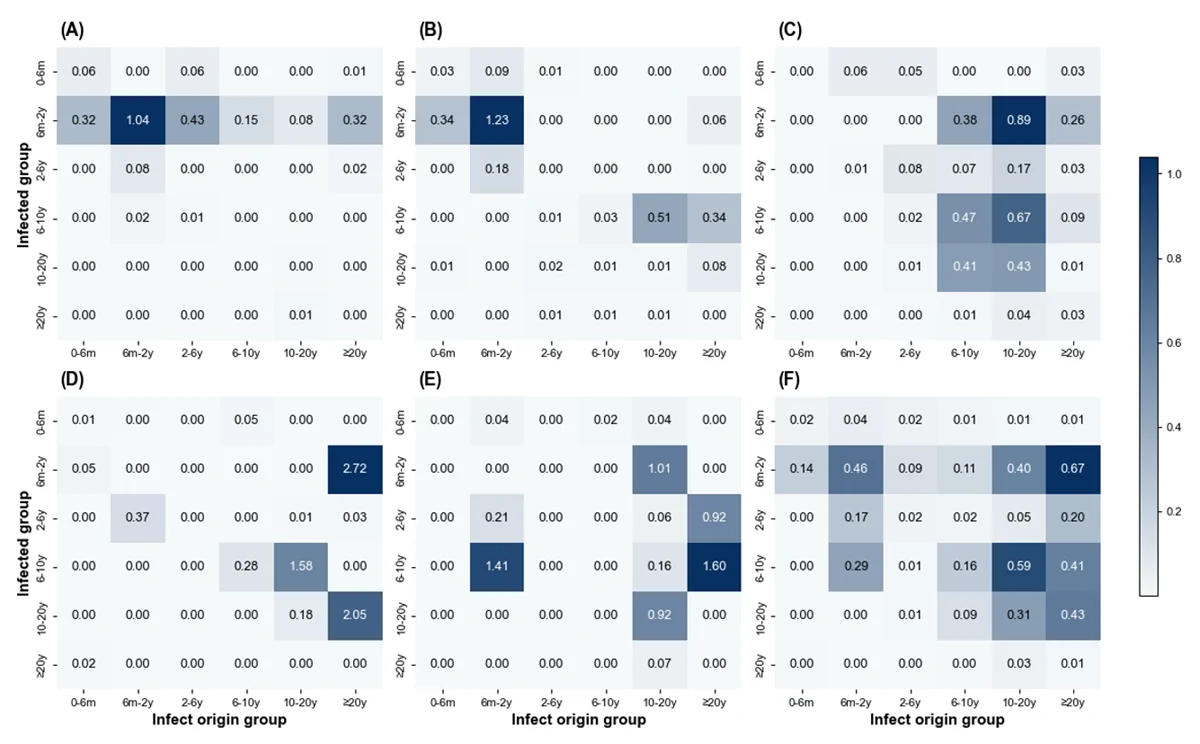
Transmissibility across age groups in Hunan Province, 2010-2025. (A) Age-structured NGMnext-generation matrix for phasePhase 1 (2010-2017); (B) phasePhase 2 (2018-2019); (C) phasePhase 3 (2020-2023); (D) phasePhase 4 (2024); (E) phasePhase 5 (2025); and (F) overall matrix for the full study period (2010-2025). In each panel, the horizontal axis represents the age group of infection origin and the vertical axis represents the age group of infected individuals. Each cell value represents the expected number of secondary cases in the recipient age group generated by one infectious individual in the source age group. Color intensity reflects transmission magnitude, with darker shading indicating higher values. The effective reproduction number ("R" _"eff" ) for each phase is given by the spectral radius of the corresponding matrix.

### Simulation of Vaccination Intervention Effectiveness

Monthly case projections (Figure S5 in Multimedia Appendix 1) showed that increasing booster coverage progressively reduced incidence peaks across all age groups. The most pronounced direct effect was observed in the 6-10 years age group, where 100% coverage reduced the projected monthly peak from 744 to 409 cases. Notably, the 0-6 months age group, ineligible for the booster, showed a monthly peak reduction from 591 to 420 cases under 100% coverage, indicating substantial indirect protection.

Annual incidence projections showed a consistent epidemic cycle across all vaccination scenarios, with incidence peaking in 2030 (Figure 5A). Under the no-booster scenario, the projected annual peak reached 24,734 cases. Increasing booster coverage progressively reduced the projected epidemic peak to 22,346, 20,803, 19,306, 18,576, 18,217, 17,861, 17,508, 17,229 and 17,160 cases at coverage levels of 30%, 50%, 70%, 80%, 85%, 90%, 95%, 99%, and 100%, respectively, corresponding to peak reductions of 9.65% (2387.90/24,734.53), 15.89% (3931.43/24,734.53), 21.95% (5428.48/24,734.53), 24.90% (6157.73/24,734.53), 26.35% (6517.24/24,734.53), 27.79% (6873.23/24,734.53), 29.21% (7225.64/24,734.53), 30.34% (7504.94/24,734.53), and 30.62% (7574.39/24,734.53).

Projected intervention effectiveness increased with both booster coverage and follow-up duration (Figure 5B). Over a 3-year horizon, cumulative reported cases were reduced by 4.25% (836.34/19,670.07), 6.98% (1372.89/19,670.07), 9.62% (1893.13/19,670.07), 10.92% (2147.27/19,670.07), 11.55% (2272.87/19,670.07), 12.19%(2397.49/19,670.07), 12.82% (2521.14/19,670.07), 13.32% (2619.37/19,670.07), and 13.44% (2643.84/19,670.07) under coverage levels of 30%, 50%, 70%, 80%, 85%, 90%, 95%, 99%, and 100%, respectively. Over a 10-year horizon, the corresponding reductions increased to 7.20% (7449.55/103,408.95), 12.04% (12,451.80/103,408.95), 16.89% (17,466.73/103,408.95), 19.32% (19,973.50/103,408.95), 20.53% (21,225.61/103,408.95), 21.74% (22,476.40/103,408.95), 22.94% (23,725.52/103,408.95), 23.91% (24,723.35/103,408.95), and 24.15% (24,972.58/103,408.95). Compared with the no-booster scenario, the projected number of averted cases over 10 years ranged from 7449 at 30% coverage to 24,972 at 100% coverage.

Age-specific analyses demonstrated substantial heterogeneity in intervention effects (Figure 5C). The largest reduction was observed in the vaccinated 6-10 years age group, where monthly peak incidence decreased by 45.06% (335.49/744.51) under the 100% coverage scenario, representing the direct effect of the booster strategy. Indirect protection was also observed in younger children, particularly among infants aged 0-6 months, in whom monthly peak incidence decreased by 28.91% (170.92/591.19). Reductions in monthly peak incidence in the remaining age groups ranged from 27.01% (233.93/865.97) to 28.52% (286.68/1005.08).

**Figure 5:**
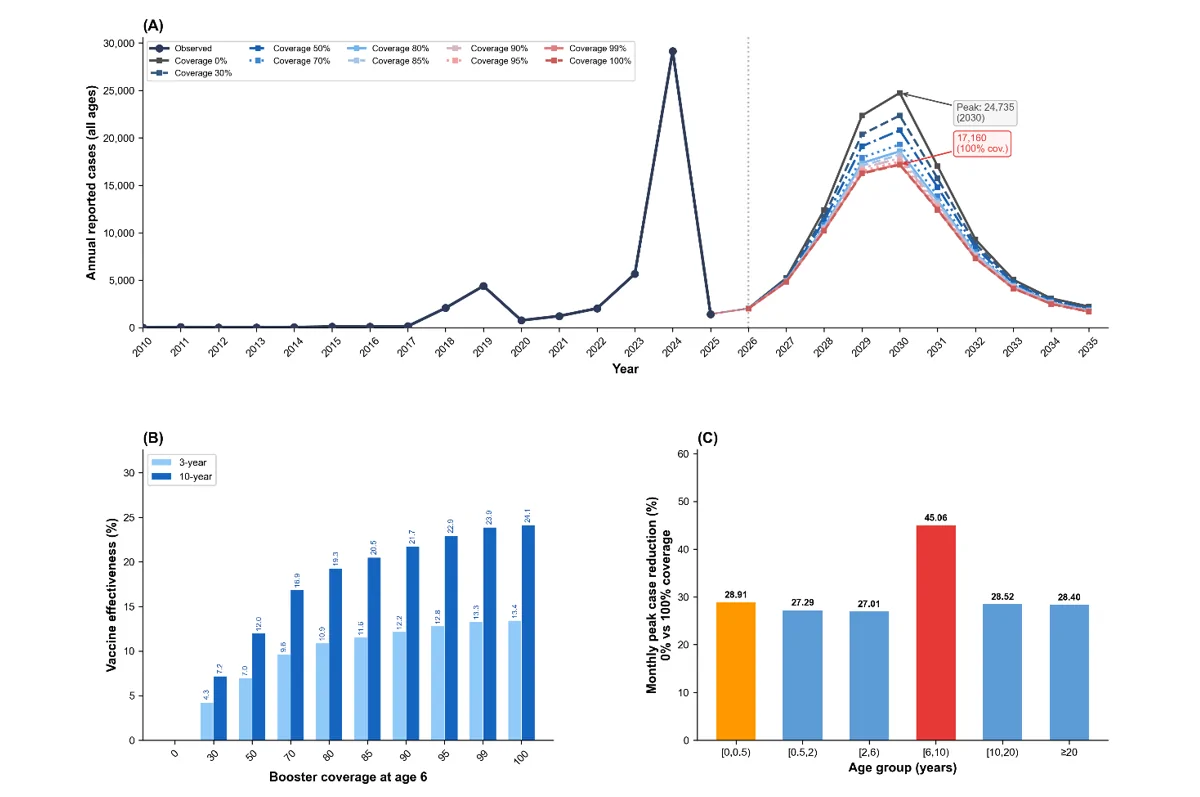
Annual predicted pertussis incidence and intervention effectiveness under gradient booster coverage. (A) Annual observed cases (2010-2025) and projected cases (2026-2035) under different vaccination coverage scenarios. (B) Effectiveness by vaccination coverage level and time horizon. (C) Age-specific peak reduction under 100% coverage, where red bars indicate the directly targeted age group, and orange and blue bars represent indirect protection, with orange indicating the most pronounced indirect effect. The vertical dashed line separates the observation period from the projection period.

### Sensitivity Analysis

Varying natural immunity duration from 5 to 20 years produced minimal changes in projected effectiveness within the same vaccine immunity scenario. Across the evaluated vaccine efficacy range of 71%-95%, projected effectiveness under 70% coverage ranged from 14.3% (range 8.0%-21.7%) to 19% (range 10.7%-28.4; Figure S6A in Multimedia Appendix 1). In contrast, under the 70% coverage scenario, the projected 10-year reduction in cumulative cases increased from approximately 9.5% (range 8.0%-11.1%) to 25% (range 21.1%-28.4%) as vaccine-derived immunity duration increased from 3 to 10 years (Figure S6B in Multimedia Appendix 1). Age-specific analyses showed the largest reduction in the 6-10 years age group, with a mean reduction of 26.5% (range 15.9%-39.4%), while the 0-6 months age group showed a reduction of 16.3% (range 7.2%-28.4%; Figure S6C in Multimedia Appendix 1). Sensitivity analyses showed that projected intervention effectiveness was most sensitive to assumptions regarding vaccine-derived immunity duration (Figure S6D in Multimedia Appendix 1).

To quantify the uncertainty of the projected outcomes, the parameter sets obtained from the parametric bootstrap were propagated through the projection model. The projected monthly reported cases under the 70% booster coverage scenario and the current 18-month booster coverage are shown in Figure S4A in Multimedia Appendix 1 with the bootstrap median and the corresponding 95% CI. The projected cumulative reductions in reported cases over 3-year and 10-year horizons increased monotonically with booster coverage, with the medians and 95% CIs summarized in Figure S4B and Table S4 in Multimedia Appendix 1. Specifically, the median 10-year reduction was 20% (IQR 15.5%-34.5%; 95% CI 11.3%-65.1%) at 70% coverage and 27.8% (IQR 22.3%-45.9%; 95% CI 15.8%-74.1%) at 100% coverage.

## Discussion

### Principal Findings

Based on surveillance data from Hunan Province between 2010 and 2025, this study identified clear phase-dependent changes in pertussis epidemiology, including temporal trends, seasonal patterns, age distribution, and transmission dynamics. The epidemic trajectory was characterized by a prolonged low-incidence period (2010-2017), a moderate increase (2018-2019), a decline during the COVID-19 period (2020-2022), and a marked resurgence in 2023-2024, followed by a decline in 2025. This pattern is consistent with recent global observations [33].

During the COVID-19 pandemic, NPIs such as social distancing, mask use, and school closures substantially reduced opportunities for the transmission of respiratory pathogens, leading to a marked decline in reported pertussis cases in many countries [22,34,35]. With the gradual relaxation of these measures, population contact rates rebounded, while reduced natural infection during the pandemic period led to the accumulation of susceptible individuals. Consequently, pertussis incidence increased significantly after 2023. This phenomenon has been described as the “immunity debt” effect [36], whereby reduced pathogen exposure in a low-transmission environment results in diminished population-level immunity, facilitating epidemic rebound after the lifting of control measures. Nationwide, approximately 0.48 million pertussis cases were reported in 2024 [33], indicating a large-scale resurgence compared to previous years. In addition, waning vaccine-induced immunity and potential genetic variation of *Bordetella pertussis* may have further contributed to the observed increase in incidence [28].

It is also possible that part of the sharp increase in reported incidence, from 0.75 per 100,000 in 2017 to 33.37 per 100,000 in 2024, reflects improvements in surveillance and diagnostic capacity rather than a true rise in transmission. In 2019, the national diagnostic criteria (WS 274-2019) were revised to formally incorporate PCR-based laboratory confirmation [3,8], and the increasing availability of PCR and serological testing over the study period may have enhanced the detection of mild and atypical cases, particularly among older children and adults. Several considerations nonetheless suggest that the resurgence was largely genuine. Reporting rates were estimated separately for each phase, so that gradual shifts in surveillance sensitivity would be absorbed into the reporting-rate parameters rather than into the estimated transmission rates, and the phase boundaries were independently supported by a data-driven change-point analysis. In addition, the 2024 surge coincided with a large-scale pertussis resurgence observed in many countries during 2023-2024, and the shift in the age distribution of cases remained robust under alternative ascertainment assumptions in our sensitivity analyses [19,37]. The absolute magnitude of the increase should therefore be interpreted with caution; its timing, direction, and accompanying age shift nevertheless appear to reflect genuine changes in pertussis epidemiology.

Our findings demonstrated a stable and consistent seasonal pattern throughout the study period, with peak incidence during spring and a secondary peak in summer, and lowest transmission during autumn. This seasonal signature persisted despite substantial changes in overall incidence magnitude and age distribution, suggesting that climatic and behavioral factors exert a consistent influence on pertussis transmission dynamics in Hunan Province. Similar seasonal patterns have been reported in multiple regions [38]. Increased indoor crowding during colder months and elevated contact rates associated with school terms may create favorable conditions for transmission [39,40].

Age-specific analysis revealed a substantial shift in case distribution over time. In the early study period (2010-2017), cases were predominantly concentrated among infants and young children [41], with the 0-6 months group accounting for 53.07% of cases. However, the proportion of cases among school-age children and adolescents has progressively increased over time. This trend is consistent with observations from other countries [42]. Infants remain the most vulnerable population because of their immature immune systems and incomplete vaccination status. The increasing proportion of cases among older children and adolescents may be attributable to the more rapid waning of immunity induced by acellular pertussis vaccines, as well as pathogen evolution affecting vaccine-induced protection. The accumulation of susceptible individuals during the COVID-19 pandemic may have further amplified these trends [10].

Our results demonstrate clear temporal shifts in the age-specific transmission dynamics. During the early phase (2010-2017), transmission was primarily concentrated within younger age groups, with infants and preschool children forming the core transmission networks. In the subsequent period (2018-2019), transmission within younger age groups further intensified, while transmission involving school-age children began to increase, indicating an expansion of the transmission network across broader age strata. During the COVID-19 period (2020-2023), transmission among younger age groups declined markedly, whereas relative transmission among older age groups increased. This shift is likely attributable to changes in contact patterns induced by NPIs [43]. Following the relaxation of these measures, a substantial resurgence occurred in 2024 [44], during which the relative contribution of older age groups increased significantly. Notably, transmission pathways from adults to children exhibited a high estimated transmission potential [45], though these estimates should be interpreted with caution given that adult cases are systematically underascertained; sensitivity analyses incorporating age-graded detection corrections suggested that the absolute magnitude of adult transmission contributions may be attenuated under higher underreporting assumptions, while the directional shift toward older age groups remained consistent across scenarios.

By 2025, the transmission structure evolved further, with adults and infants emerging as 2 key nodes in the transmission network, and cross-age transmission played a more prominent role in sustaining the epidemic spread. This is consistent with the well-documented role of household transmission, in which parents and older siblings are frequently the source of infection for unprotected infants [5,46-49]. Because immunity induced by acellular vaccines wanes within a few years, many adults and adolescents experience mild or atypical infection that is frequently unrecognized and untreated; they nevertheless shed *Bordetella pertussis* and can transmit to susceptible household contacts [50]. In addition, school-age children, owing to their high contact rates in school settings, may act as “bridges” connecting different age groups, thereby facilitating sustained community transmission [48,51]. The rebound of social contact after the relaxation of NPIs in 2024, together with the accumulation of susceptible adolescents and adults during the pandemic, plausibly amplified these cross-age pathways and contributed to the age-shift observed in the present study.

Notably, the ≥20 years group contributed only 1597 (3.4%) of the 47,300 reported cases over the study period, ranking lowest among all age groups; given its considerably larger population size, its reported incidence was correspondingly low. This sparsity largely reflects underascertainment rather than a true absence of transmission: adults are less likely to seek medical care, undergo diagnostic testing, or be captured by surveillance when presenting with mild disease. The sparse and 0-inflated reporting in this group therefore cannot represent its true transmission potential, and further subdivision into adult and older adult subgroups would yield case series dominated by 0 counts that cannot support reliable estimation. The ≥20 years group was accordingly retained as a single category, and estimates involving this group should be interpreted with caution; nevertheless, the direction of the age shift—increasing transmission from older age groups—remains robust.

Although a DTaP booster at the age of 6 years has only recently been introduced in China, immunogenicity studies in 6-year-old Chinese children have demonstrated robust serological responses following an additional DTaP dose, with seroconversion rates exceeding 80% for key pertussis antigens [52]. Furthermore, the well-documented waning of protection following the fifth DTaP dose [53], combined with the disproportionate burden of pertussis among school-age children in China [54], provides a strong epidemiological rationale for targeting this age group with a booster dose. The simulation results support the epidemiological rationale for a booster dose at the age of 6 years as a strategic intervention to disrupt cross-age transmission. At 100% coverage, the 6-10 years group showed the greatest monthly peak reduction (45.06%), while vaccine-ineligible infants also benefited through indirect protection (28.91% peak reduction). This pattern of herd protection is particularly important given that infants below 6 months carry the highest risk of severe pertussis and are too young to complete primary vaccination [55]. The surveillance dataset used in this study contains aggregated, age-stratified case counts without clinical severity indicators, so temporal trends in infant disease severity cannot be quantified directly from these data. Nevertheless, severe and fatal pertussis has remained rare and concentrated among unvaccinated or incompletely vaccinated young infants since the nationwide introduction of routine pertussis vaccination in 1978, and the DTwP-to-DTaP transition did not alter this pattern. Most cases in older age groups are mild and recover with appropriate treatment [56]. Marginal effectiveness analysis indicated that achieving and sustaining coverage at or above 70% should be the priority public health target, as this threshold captures the majority of achievable benefit. The temporal dynamics of effectiveness further demonstrate that the value of the booster strategy is realized progressively over time. Nevertheless, even at 100% coverage, the projected 10-year effectiveness of 24.1% indicates that a single-age booster alone cannot fully interrupt transmission [57]. Complementary strategies, such as booster doses of Tdap (Tetanus, Diphtheria, and Acellular Pertussis) vaccine for boosters for adolescents and adults and maternal immunization during pregnancy, warrant integration into future immunization policy frameworks [58-60].

### Limitation

Several limitations should be acknowledged. First, phases 4 and 5 each comprise only 13-15 monthly observations, which limited the identifiability of the individual age-specific transmission coefficients in these phases, as reflected in the flat directions of the fitting objective and the wide bootstrap intervals for individual parameters. Our conclusions therefore rely primarily on aggregate transmission measures, the effective reproduction number, and projected outcomes, for which bootstrap CIs are reported; the qualitative finding that higher booster coverage confers a greater reduction in reported cases remained consistent across the bootstrap replicates. Second, surveillance-based pertussis data are likely affected by age-dependent underreporting, particularly among adolescents and adults who often present with mild or atypical symptoms. Although sensitivity analyses showed that the overall transmission pattern remained stable across different ascertainment assumptions, the absolute magnitude of transmission estimates may still be uncertain. Third, several model parameters, including immunity duration and vaccine efficacy, were derived from published literature rather than directly estimated from local immunological data. Fourth, the projection analyses assumed stable demographic structure and contact patterns over time and did not incorporate behavioral changes, pathogen evolution, or future modifications to vaccination policy. Finally, the study was based on data from a single province and may not fully represent transmission dynamics in other regions of China.

### Conclusions

This study demonstrated a progressive shift in pertussis transmission toward older age groups, with increasing cross-age transmission to younger children and infants. Simulation analyses suggested that introduction of a booster dose at the age of 6 years could reduce overall pertussis incidence and provide indirect protection to infants, although the projected impact of a single school-age booster alone remained moderate. These findings provide quantitative evidence for optimizing age-targeted pertussis vaccination strategies in China.

## Appendix

Multimedia Appendix 1 Data showing validation of the study phases, sex-stratified case distributions, segmented model fitting and parameter identifiability, and bootstrap and sensitivity analyses of the projected impact of the 6-year booster dose.

Multimedia Appendix 2 STROBE checklist.

## Abbreviations

DTaP: Diphtheria, Tetanus Toxoids, and Acellular Pertussis Vaccine
DTwP: Diphtheria, Tetanus, and Whole-Cell Pertussis Vaccine
NGM: next-generation matrix
NPI: nonpharmaceutical intervention
PCR: polymerase chain reaction
STROBE: Strengthening the Reporting of Observational Studies in Epidemiology
Tdap: Tetanus, Diphtheria, and Acellular Pertussis
VSEIR: Vaccinated-Susceptible-Exposed-Infectious-Recovered

## Data Availability

The aggregated surveillance data supporting the findings of this study are available from the corresponding author upon reasonable request and with permission from the Hunan Provincial Center for Disease Control and Prevention. Restrictions apply to the availability of individual-level surveillance data because of privacy regulations. The source code used for model fitting, simulation, and figure generation is publicly available in a GitHub repository [61].

## References

[1] Torres RSLA, Santos TZ, Torres RAA, Pereira VVG, Fávero LAF, M Filho OR, Penkal ML, Araujo LS (2015). Resurgence of pertussis at the age of vaccination: clinical, epidemiological, and molecular aspects. J Pediatr (Rio J).

[2] Kretzschmar M, Teunis PFM, Pebody RG (2010). Incidence and reproduction numbers of pertussis: estimates from serological and social contact data in five European countries. PLoS Med.

[3] Pediatric Infection Group‚ Chinese Society of Infectious Diseases‚ Chinese Medical Association, Infection Group‚ Pediatric Expert Committee of National Health Commission Capacity BuildingContinuing Education, China Clinical Practice Guidelines Alliance Methodology Committee, National Children′s Medical Center (Shanghai), National Medical Center for Infectious Diseases (2024). Guidelines for diagnosis and management and prevention of pertussis of China (2024 edition). Zhonghua Yi Xue Za Zhi.

[4] Xu S, Yuan L, Liu W, Ye C, Li Z, Segbo JAG, Jin Z, Zhu C (2025). Epidemiological and clinical characteristics of pertussis in pediatric population: a multicenter study of 20,727 children in Hubei, China. BMC Infect Dis.

[5] Decker MD, Edwards KM (2021). Pertussis (whooping cough). J Infect Dis.

[6] Zheng S, Luo F, Su Y, Liu X, Dai R, Deng X, Zhu Y, Zhou Y, He H, Hua C, Zhang H (2025). Clinical characteristics and mortality risk factors among hospitalized infants and children with pertussis in China: a retrospective study. Front Public Health.

[7] de Cellès MD, Rohani P (2024). Pertussis vaccines, epidemiology and evolution. Nat Rev Microbiol.

[8] Pertussis [in Chinese]. Chinese Center for Disease Control and Prevention.

[9] Gorringe A, Cavell B, Beard F, Tsukada K, Otsuka N, Fu P, Moosa F, Fabianova K, Rodrigues C, Bouchez V, Toubiana J, Brisse S, Dalby T, He Q, Campbell H, Hozbor D, Hariri S, Pawloski L, Scanlon K, Edwards K (2025). Global incidence of pertussis after the COVID-19 pandemic. JAMA Netw Open.

[10] Wan M, Zhang G, Yi H (2024). Unraveling the resurgence of pertussis: insights into epidemiology and global health strategies. Pulmonology.

[11] Chen Z, He Q (2017). Immune persistence after pertussis vaccination. Hum Vaccines Immunother.

[12] Gao H, Lau EHY, Cowling BJ (2022). Waning immunity after receipt of pertussis, diphtheria, tetanus, and polio-related vaccines: a systematic review and meta-analysis. J Infect Dis.

[13] Schwartz KL, Kwong JC, Deeks SL, Campitelli MA, Jamieson FB, Marchand-Austin A, Stukel TA, Rosella L, Daneman N, Bolotin S, Drews SJ, Rilkoff H, Crowcroft NS (2016). Effectiveness of pertussis vaccination and duration of immunity. CMAJ.

[14] Wu D, Jing R, Zheng H, He K, Li Y, Yu W, Yin Z, Fang H (2023). Health and economic evaluation of vaccination against pertussis in China: a 40-year analysis. Value Health.

[15] (2024). Notice on adjusting the immunization program for diphtheria-pertussis-tetanus vaccine and diphtheria-tetanus vaccine under the national immunization program. Prevention CsNCfDCa.

[16] Rozenbaum MH, De Vries R, Postma MJ (2012). Modelling the impact of extended vaccination strategies on the epidemiology of pertussis. Epidemiol Infect.

[17] Choi YH, Campbell H, Amirthalingam G, van Hoek AJ, Miller E (2016). Investigating the pertussis resurgence in England and Wales, and options for future control. BMC Med.

[18] Qiao Y, Zhao J (2026). Study on the resurgence of pertussis based on a stage-structured dynamic model. Infect Dis Model.

[19] Yang Q, Yang Y, Liu B, Xu L, Ma Y, Zhou J, Wang Y, Hao C, Jiang W (2026). Global perspectives on pertussis epidemiology, macrolide resistance, and management in the post-COVID-19 era (2020-2024). J Microbiol Immunol Infect.

[20] Bento AI, Rohani P (2016). Forecasting epidemiological consequences of maternal immunization. Clin Infect Dis.

[21] Subspecialty Group of Infectious Diseases‚ the Society of Pediatrics‚ Chinese Medical Association, Editorial Board‚ Chinese Journal of Pediatrics (2017). Recommendation for diagnosis and treatment of Chinese children with pertussis. Zhonghua Er Ke Za Zhi.

[22] Zhou J, Sun Y, Shen H, Yang L, Ye Q, Zhu H, Zhu Z (2025). Impact of COVID-19 and nonpharmaceutical interventions on common respiratory viruses in children: a 5-year study in Hangzhou, China. Intervirology.

[23] Hunan Provincial Bureau of Statistics (2025). Hunan Statistical Yearbook.

[24] US Centers for Disease Control and Prevention (2024). Chapter 16: pertussis. Epidemiology and Prevention of Vaccine-Preventable Diseases.

[25] Finger H, von Koenig CHW (1996). Bordetella. Medical Microbiology.

[26] Wilkinson K, Righolt CH, Elliott LJ, Fanella S, Mahmud SM (2021). Pertussis vaccine effectiveness and duration of protection - a systematic review and meta-analysis. Vaccine.

[27] Zhu P, Wu D, Wang Y, Liu X, Rodewald LE, Li Y, Zheng H, Cao L, Song Y, Song L, Zhao X, Yao J, Wang F, Li M, Zhang Q, Yan T, Yin Z (2023). Effectiveness of DTaP against pertussis in ≤2-Year-old children - Linyi prefecture, Shandong Province, China, 2017-2019. China CDC Wkly.

[28] Gambhir M, Clark TA, Cauchemez S, Tartof SY, Swerdlow DL, Ferguson NM (2015). A change in vaccine efficacy and duration of protection explains recent rises in pertussis incidence in the United States. PLoS Comput Biol.

[29] Chit A, Zivaripiran H, Shin T, Lee JKH, Tomovici A, Macina D, Johnson DR, Decker MD, Wu J (2018). Acellular pertussis vaccines effectiveness over time: a systematic review, meta-analysis and modeling study. PLoS One.

[30] Zhang H, Lai X, Mak J, Sriudomporn S, Zhang H, Fang H, Patenaude B (2022). Coverage and equity of childhood vaccines in China. JAMA Netw Open.

[31] Wendelboe AM, Van Rie A, Salmaso S, Englund JA (2005). Duration of immunity against pertussis after natural infection or vaccination. Pediatr Infect Dis J.

[32] Diekmann O, Heesterbeek JAP, Roberts MG (2010). The construction of next-generation matrices for compartmental epidemic models. J R Soc Interface.

[33] (2023). Annual epidemiological report. European Centre for Disease Prevention and Control.

[34] Li K, Rui J, Song W, Luo L, Zhao Y, Qu H, Liu H, Wei H, Zhang R, Abudunaibi B, Wang Y, Zhou Z, Xiang T, Chen T (2024). Temporal shifts in 24 notifiable infectious diseases in China before and during the COVID-19 pandemic. Nat Commun.

[35] Kang HM, Lee T, Park SE, Choi S (2025). Pertussis in the post-COVID-19 era: resurgence, diagnosis, and management. Infect Chemother.

[36] Munro APS, House T (2026). Cycles of susceptibility: immunity debt explains altered infectious disease dynamics post-pandemic. Clin Infect Dis.

[37] Rodrigues C, Bouchez V, Soares A, Trombert-Paolantoni S, Aït El Belghiti F, Cohen JF, Armatys N, Landier A, Blanchot T, Hervo M, Toubiana J, Brisse S, REMICOQ study group (2024). Resurgence of Bordetella pertussis, including one macrolide-resistant isolate, France, 2024. Euro Surveill.

[38] Wang Y, Xu C, Ren J, Zhao Y, Li Y, Wang L, Yao S (2020). The long-term effects of meteorological parameters on pertussis infections in Chongqing, China, 2004-2018. Sci Rep.

[39] Huang X, Lambert S, Lau C, Soares Magalhaes RJ, Marquess J, Rajmokan M, Milinovich G, Hu W (2017). Assessing the social and environmental determinants of pertussis epidemics in Queensland, Australia: a Bayesian spatio-temporal analysis. Epidemiol Infect.

[40] De Greeff SC, Dekkers ALM, Teunis P, Rahamat-Langendoen JC, Mooi FR, De Melker HE (2009). Seasonal patterns in time series of pertussis. Epidemiol Infect.

[41] Nie Y, Zhang Y, Yang Z, Wang N, Wang S, Liu Y, Jiang H, Wu L (2024). Global burden of pertussis in 204 countries and territories, from 1990 to 2019: results from the Global Burden of Disease Study 2019. BMC Public Health.

[42] Paireau J, Guillot S, El Belghiti FA, Matczak S, Trombert-Paolantoni S, Jacomo V, Taha M, Salje H, Brisse S, Lévy-Bruhl D, Cauchemez S, Toubiana J (2022). Effect of change in vaccine schedule on pertussis epidemiology in France: a modelling and serological study. Lancet Infect Dis.

[43] Wu N, Guan P, An S, Wang Z, Huang D, Ren Y, Wu W (2024). Assessing the impact of COVID-19 non-pharmaceutical interventions and relaxation policies on class B respiratory infectious diseases transmission in China. Sci Rep.

[44] Reicherz F, Li S, Watts AA, Goldfarb DM, Lavoie PM, Abu-Raya B (2024). Bordetella pertussis infection following relaxation of COVID-19 non-pharmaceutical interventions in 2021-2023 in Vancouver metropolitan area, British Columbia, Canada. Vaccine.

[45] Arakkal AT, Cavanaugh JE, Polgreen PM, Miller AC (2024). The increase in household transmission of pertussis associated with diagnostic delays. Pediatr Infect Dis J.

[46] Kara EO, Campbell H, Ribeiro S, Fry NK, Litt D, Eletu S, Amirthalingam G (2017). Survey of household contacts of infants with laboratory-confirmed pertussis infection during a national pertussis outbreak in England and Wales. Pediatr Infect Dis J.

[47] Su Y, Dai R, Luo F, Zheng S, Hua C, He H, Zhang H (2024). Household transmission patterns and serial interval of pertussis in China. J Infect.

[48] Luo F, Zheng S, Zhou Y, Zhu Y, Su Y, Dai R, Hua C, Zhang H, He H (2025). Sources and clinical characteristics of infant pertussis in China: increasing contribution of siblings. BMC Infect Dis.

[49] Wendelboe AM, Njamkepo E, Bourillon A, Floret DD, Gaudelus J, Gerber M, Grimprel E, Greenberg D, Halperin S, Liese J, Muñoz-Rivas F, Teyssou R, Guiso N, Van Rie A, Infant Pertussis Study Group (2007). Transmission of Bordetella pertussis to young infants. Pediatr Infect Dis J.

[50] Tartof SY, Lewis M, Kenyon C, White K, Osborn A, Liko J, Zell E, Martin S, Messonnier NE, Clark TA, Skoff TH (2013). Waning immunity to pertussis following 5 doses of DTaP. Pediatrics.

[51] Wang J, Xu J, Xiao D, Li B, Xiong Y, Yang J, Wei W, Chen L, Wang Q, Qin Y, Liu Y, Zhao H, Long J, Qi L (2025). Epidemiological and spatial-temporal clustering characteristics of pertussis in Southwest China, 2013-2024. Front Public Health.

[52] Tang X, Xiao Y, Chen J, Su Y, Zhou Y, Luo L, Zhang J, Liu S, Yan R, Zhu D, Zhao W, Zhu Y, Ma X, Jiang Y, Pan H, Zhang Y, He H (2025). Immunogenicity and safety of co-purified diphtheria, tetanus and acellular pertussis vaccine in 6-year-old Chinese children. Nat Commun.

[53] Klein NP, Bartlett J, Rowhani-Rahbar A, Fireman B, Baxter R (2012). Waning protection after fifth dose of acellular pertussis vaccine in children. N Engl J Med.

[54] Liu X, Wei C, Huang H, Wan J, Li Y, Wang F, Li S, Wang Y, Wang X, Wang X, Sui X, Gou J, Zhu T, Ma X, Hu W (2025). Preliminarily evaluation the safety and immunogenicity of tetanus, reduced diphtheria and acellular pertussis (five components) combined vaccine, adsorbed (Tdcp) in participants aged 6 years and above: a blinded and randomised, and controlled phase I clinical trial. Front Immunol.

[55] Sun X (2025). Dilemma in prevention of pertussis infection among infants under six months in China. Expert Rev Vaccines.

[56] Hua CZ, He HQ, Shu Q (2024). Resurgence of pertussis: reasons and coping strategies. World J Pediatr.

[57] Riolo MA, Rohani P (2015). Combating pertussis resurgence: one booster vaccination schedule does not fit all. Proc Natl Acad Sci U S A.

[58] Shi Q, Li J, Hu Q, Cheng C, Yang K, Li X, Song X, Chen S, Duan G (2025). Efficacy, immunogenicity, and safety of pertussis vaccine during pregnancy: a meta-analysis. Vaccines (Basel).

[59] Regan AK, Moore HC, Binks MJ, McHugh L, Blyth CC, Pereira G, Lust K, Sarna M, Andrews R, Foo D, Effler PV, Lambert S, Van Buynder P (2023). Maternal pertussis vaccination, infant immunization, and risk of pertussis. Pediatrics.

[60] Versteegen P, Marinović AAB, van Gageldonk PGM, van der Lee S, Hendrikx LH, Sanders EAM, Berbers GAM, Buisman A (2022). Long-term immunogenicity upon pertussis booster vaccination in young adults and children in relation to priming vaccinations in infancy. Vaccines (Basel).

[61] He Y pertussis_vaccine. GitHub.

