# Supplementary material for: Age-Specific Pertussis Transmission Dynamics and Impact of Immunization Schedule Revision in Hunan, China: Age-Structured Mathematical Modeling Study
Source: JMIR Public Health Surveill. 2026 Oct 27;12:e104376. doi: 10.2196/104376 (PMC13752597; doi:10.2196/104376)
Supplement: Supplementary file 1 [file publichealth_v12i1e104376_app1.docx]

**Figure S1.** Change-point consistency analysis of the age-specific monthly reported pertussis cases. (A)-(E) Change-point detection results for age groups 1-5, respectively. In each panel, the black line shows the observed monthly case counts; the red dashed vertical lines indicate the segment boundaries used in the main analysis (months 96, 120, and 166); and the dotted vertical lines denote the change points detected by the PELT (red), CUSUM (teal), and BIC (purple) methods, respectively.

**Table S1** Consistency between the segment boundaries used in the model and the change points independently detected by three methods in each age group, within a tolerance of ±6 months.

| **Age group** | **PELT** | **CUSUM** | **BIC** |
| --- | --- | --- | --- |
| 1 | 3/4 | 0/4 | 2/4 |
| 2 | 4/4 | 0/4 | 2/4 |
| 3 | 3/4 | 2/4 | 2/4 |
| 4 | 2/4 | 2/4 | 0/4 |
| 5 | 2/4 | 1/4 | 0/4 |
| 6 | 2/4 | 1/4 | 1/4 |
| Overall | 16/24 (66.7%) | 6/24 (25.0%) | 7/24 (29.2%) |

**Figure S2.** Sex-stratified descriptive analysis of reported pertussis cases in Hunan Province, China, 2010-2025.

(A) Monthly reported cases by sex (blue, male; red, female), with the inset showing an enlarged view of the early low-incidence period (2010-2017); the arrow indicates that the inset corresponds to the shaded portion of the main curve. (B) Annual sex composition of reported cases expressed as proportions (blue, male; red, female); the dashed line marks an equal sex ratio (50%). (C) Age distribution of reported cases by sex, expressed as the percentage of cases within each sex; the male and female profiles overlap almost completely across all age groups. (D) Male-to-female case ratio by study phase and age group; cells with fewer than 30 cases (†) are based on sparse data and should be interpreted with caution.

**Table S2.** Sex-stratified descriptive statistics and statistical tests for sex differences in reported pertussis cases, Hunan Province, 2010-2025

Part A. Cases, incidence, and incidence rate ratios by study phase

| **Phase** | **Male cases** | **Female cases** | **M:F case ratio (95% CI)** | **Male incidence (per 100,000)** | **Female incidence (per 100,000)** | **Incidence rate ratio (95% CI)** | **p-value** |
| --- | --- | --- | --- | --- | --- | --- | --- |
| Overall (2010–2025) | 24,811 | 22,557 | 1.10 (1.08–1.12) | 4.51 | 4.34 | 1.04 (1.02–1.06) | <0.001 |
| Phase 1 (2010–2017) | 293 | 259 | 1.13 (0.96–1.34) | 0.83 | 0.78 | 1.06 (0.90–1.26) | 0.465 |
| Phase 2 (2018–2019) | 3,444 | 3,020 | 1.14 (1.09–1.20) | 9.64 | 9.02 | 1.07 (1.02–1.12) | 0.007 |
| Phase 3 (2020–2023) | 4,922 | 4,767 | 1.03 (0.99–1.07) | 14.63 | 14.88 | 0.98 (0.95–1.02) | 0.414 |
| Phase 4 (2024) | 15,366 | 13,852 | 1.11 (1.08–1.14) | 45.92 | 43.38 | 1.06 (1.03–1.08) | <0.001 |
| Phase 5 (2025) | 786 | 659 | 1.19 (1.08–1.32) | 2.35 | 2.06 | 1.14 (1.03–1.26) | 0.014 |

**Part B. Case distribution by age group and sex**

| **Age group** | **Male** | **Female** | **M:F ratio (95% CI)** |
| --- | --- | --- | --- |
| 0–6 months | 4,889 | 3,907 | 1.25 (1.20–1.31) |
| 6 months–2 years | 4,246 | 3,574 | 1.19 (1.14–1.24) |
| 2–6 years | 6,914 | 6,825 | 1.01 (0.98–1.05) |
| 6–10 years | 6,595 | 5,895 | 1.12 (1.08–1.16) |
| 10–20 years | 1,623 | 1,297 | 1.25 (1.16–1.35) |
| ≥20 years | 544 | 1,059 | 0.51 (0.46–0.57) |
| Chi-square (age × sex) | χ² = 302.47, df = 5, p < 0.001 |  | Cramér's V = 0.08 |

**Part C. Age at onset and adjusted sex effect**

| **Measure** | **Male** | **Female** | **Test result** |
| --- | --- | --- | --- |
| Age at onset (months), median (IQR) | 48 (8–72) | 48 (11–84) | Mann–Whitney U p < 0.001; rank-biserial r = 0.04 |
| Monthly case counts, negative-binomial regression (sex effect) | — | — | IRR = 1.07 (95% CI 0.86–1.34), p = 0.543 |

**Figure S3.** Segmented model fitting results for pertussis cases across age groups in Hunan Province, 2010–2025. (A) 0-6 months; (B) 6 months-2 years; (C) 2-6 years; (D) 6-10 years; (E) 10-20 years; (F) ≥20 years. The dashed lines represent observed monthly cases, and the solid lines represent model-fitted values. Vertical dotted lines indicate phase boundaries used for segmented model fitting.

**Table S3.** Local identifiability diagnostics and bootstrap uncertainty of transmission and reporting parameters for 2024-2025

| Item | Phase 4 | Phase 5 |
| --- | --- | --- |
| Number of observations and parameters | 90/42 | 78/42 |
| Condition number of J′J | 3.6×10¹³ | 2.9×10¹⁴ |
| Near-zero eigenvalues | 29/42 | 19/42 |
| Maximum \|r\| (*β*-*β*) | 0.978 | 0.999 |
| Proportion of *β* at lower bound (bootstrap) | 46.6% | 66.3% |
| Proportion of *β* at upper bound | 3.6% | 7.6% |
| Proportion of ρ at lower bound | 10.0% | 25.0% |
| Median *R_eff_* [95% CI] | 1.24 [0.92-1.86] | 1.15[0.73-1.40] |
| Mean *β* [95% CI] | 0.283 [0.185-0.422] | 0.297[0.135-0.449] |
| S-weighted *β* [95% CI] | 1.588 [1.227-2.401] | 1.921[0.788-2.992] |

**Table S4.** Bootstrap 95% confidence intervals for the predicted cumulative reduction in reported cases by 6-year booster coverage

| Coverageof 6-year booster  (%) | 10-year reduction in reported cases (%) | | | 3-year reduction in reported cases (%) | | |
| --- | --- | --- | --- | --- | --- | --- |
|  | Median | 95%CI lower | 95%CI upper | Median | 95%CI lower | 95%CI upper |
| 30 | 8.73 | 4.94 | 36.95 | 4.92 | 2.5 | 11.09 |
| 50 | 14.46 | 8.16 | 54.18 | 7.92 | 4.12 | 17.38 |
| 70 | 19.97 | 11.27 | 65.07 | 10.58 | 5.71 | 22.94 |
| 80 | 22.67 | 12.79 | 68.8 | 11.99 | 6.5 | 25.49 |
| 85 | 23.78 | 13.54 | 70.35 | 12.68 | 6.89 | 26.7 |
| 90 | 25.09 | 14.28 | 71.74 | 13.37 | 7.27 | 27.88 |
| 95 | 26.39 | 15.02 | 72.99 | 14.05 | 7.66 | 29.03 |
| 99 | 27.48 | 15.61 | 73.89 | 14.59 | 7.96 | 29.92 |
| 100 | 27.76 | 15.76 | 74.11 | 14.72 | 8.04 | 30.14 |

**Figure S4.** Bootstrap uncertainty analysis of the projected effectiveness of the 6-year pertussis booster strategy. (A) Projected monthly reported cases of pertussis under 70% booster coverage over the 10-year projection horizon, showing the point prediction using the original fitted parameters (solid line), the median of the 30 parametric bootstrap replicates (dashed line), and the corresponding 95% confidence interval (shaded band); (B) Projected cumulative reduction in reported pertussis cases over 3-year and 10-year horizons under different booster coverage levels, with medians and 95% confidence intervals derived from the parametric bootstrap.

**Figure S5.** Monthly predicted pertussis cases by age group under gradient vaccination coverage, 2026-2035. (A) 0-6 months; (B) 6 months-2 years; (C) 2-6 years; (D) 6-10 years; (E) 10-20 years; (F) ≥20 years. Lines represent observed cases, model-fitted values, and projections under different booster coverage levels (0%, 30%, 50%, 70%, 80%, 85%, 90%, 95%, 99% and 100%). The vertical dashed line indicates the boundary between the calibration period (before 2025) and the projection period (2026-2035).

**Figure S6.** Sensitivity analyses of projected effectiveness of the 6-year pertussis booster strategy. (A) Influence of vaccine-derived immunity duration (EPS1) and natural immunity duration (EPS2) on projected intervention effectiveness under 70% booster coverage for 10 years; (B) Projected intervention effectiveness across different booster coverage levels and vaccine efficacy assumptions for 10 years; (C) Age-specific projected effectiveness under different booster coverage levels, including direct protection in the 6-10 years age group and indirect protection in infants aged 0-6 months; (D) Tornado analysis showing the relative influence of vaccine-derived immunity duration, intervention efficacy, and natural immunity duration on projected intervention effectiveness under 70% booster coverage for 10 years.
